# Cervico-Vaginal Microbiome Dynamics Across HPV-Driven Lesion Stages in Moroccan Women

**DOI:** 10.3390/microorganisms13081884

**Published:** 2025-08-13

**Authors:** Malika Allali, Khaoula Errafii, Rachid El Fermi, Karima Fichtali, Sanaa El Majjaoui, Adil El Ghanmi, Hicham El Fazazi, Najib Al Idrissi, Bouchra Ghazi, Youssef Bakri, Hassan Ghazal, Salsabil Hamdi

**Affiliations:** 1 Virology and Public Health Laboratory, Institut Pasteur du Maroc, Casablanca 20360, Morocco; allalimalika96@gmail.com; 2 Laboratory of Human Pathologies Biology, Department of Biology, Faculty of Sciences, University Mohammed V, Rabat 10000, Morocco; bakri@fsr.ac.ma; 3 African Genome Center, Mohamed IV Polytechnic University, Benguerir 43151, Morocco; rachid.elfermi@um6p.ma; 4 Immunopathology-Immunotherapy-Immunomonitoring Laboratory, Faculty of Medicine, Mohammed VI University of Health and Sciences (UM6SS), Casablanca 82403, Morocco; karima.fichtali@yahoo.fr (K.F.); drsanaamajjaoui@hotmail.com (S.E.M.); aelghanmi@um6ss.ma (A.E.G.); bghazi@um6ss.ma (B.G.); 5 Fertility Center Cheikh Zaid International University Hospital, Abulcasis International University of Health Sciences, Rabat 10000, Morocco; hichamelfazazi@gmail.com; 6 Laboratory of Genomics, Genetics, Epigenetics, Precision and Predictive Medicines (PerMed), Faculty of Medicine, Mohammed VI University of Sciences and Health, Casablanca 82403, Morocco; nalidrissi@um6ss.ma (N.A.I.); hassan.ghazal@fulbrightmail.org (H.G.); 7 Royal Institute of Managerial Training, Department of Sports Sciences, Sale 11000, Morocco

**Keywords:** cervical cancer, high-risk HPV, 16S rRNA sequencing, cervico-vaginal microbiome, microbial biomarkers

## Abstract

Cervical cancer (CC), often caused by persistent high-risk HPV infection, is a major health issue for Moroccan women. This study is the first in Morocco to examine how the cervico-vaginal microbiome differs across HPV-related clinical stages. Using 16S rRNA sequencing, the researchers analyzed samples from 247 women—100 healthy controls, 43 hr-HPV^+^ pre-cancer cases, and 104 post-treatment CC cases. In healthy women, *Lactobacillus* dominated (70%), but it significantly declined in the pre-cancer group (45%, *p* < 0.01) and remained low post-treatment (50%). Meanwhile, Pseudomonadota and Actinobacteriota increased in pre-cancer samples (up to 25–30%, *p* < 0.01). Although the alpha diversity remained stable, the beta diversity differed significantly across stages (*p* = 0.001), but not by HPV status. Post-treatment samples showed a sharp decline in Bacillota (logFC −5, *p* < 10^−15^) and increases in Campylobacterota and Fusobacteriota (logFC +6 to +21, *p* < 10^−16^). Functionally, chemo-heterotrophy and fermentation declined, while nitrogen fixation and phototrophy rose in pre-cancer cases. Host factors like late menarche, high parity, STIs, and contraceptive use correlated with specific microbiota shifts.

## 1. Introduction

Cervical cancer (CC) is the fourth most common cancer in women globally, with around 604,127 new cases and 341,831 deaths annually [1]. It is second among cancers in females in Morocco, after breast cancer [2]. About 10.4 out of every 100,000 Moroccan females develop CC each year, and 5.8 out of every 100,000 die from it [1]. The main etiology of CC is infection with human papillomavirus (HPV), and it accounts for around 99.7% of cases [3]. HPV is a DNA virus without an envelope that infects human skin and mucosal tissues. Around 10% of women affected by HPV cannot fully clear the virus, resulting in a long-term infection with high-risk HPV types (hr-HPV) [4]. Infection with high-risk HPV types is one of the main causes of CC. For example, HPV16 and HPV18 are two high-risk HPV types and cause about 55% and 15% of all CC cases, respectively. HPV16 infection is especially linked to cervical squamous cell carcinoma [5]. In Morocco, the rate of HPV infection differs greatly between geographic regions. Studies show varying infection rates, from as low as 13.3% to as high as 76% in the north–central parts of the country [2]. Additionally, practices like vaginal douching, sexually transmitted infections, vulvovaginitis, vulvitis, and bacterial vaginosis (BV) can disrupt the normal vaginal environment. These factors probably enable persistent high-risk HPV infections to develop [6,7]. Other factors associated with persistent HPV infection include older age, smoking, a weakened immune system, contraceptive use, and infection with the bacterium Chlamydia trachomatis [2,8]. Microtrauma during HPV infection can destroy the biologic barrier formed by the local vaginal immune microenvironment, leading to the multiplication of a large number of abnormal flora [9]. HPV infection is necessary but not enough on its own to cause cervical pre-cancerous lesions and invasive cancer. Over the past several years, interest has grown regarding how the vaginal and cervical microbial environment might influence the development and progression of cervical pre-cancerous conditions [10]. The vaginal microbiota is very important for women’s health. It is a sensitive and constantly changing environment that varies throughout a woman’s life [11]. Out of all microbial communities in the human body, the vaginal microbiota constitutes about 9% of the entire human microbiome [12]. Although previous studies have indicated associations between the cervico-vaginal microbiome composition and HPV infection, the detailed microbiome shifts across HPV-driven cervical lesion stages, particularly in Moroccan populations, remain poorly understood. Recent metagenomic studies have shown that the vaginal microbiota is mainly composed of Firmicutes bacteria, with smaller amounts of *Proteobacteria*, *Bacteroidetes*, *Fusobacteria*, and *Actinobacteria* [12]. It also includes fungi such as Candida and *Pichia kudriavzevii* and viruses from families like Papillomaviridae, Herpesviridae, Anelloviridae, and Polyomaviridae [13,14]. *Lactobacillus* species are the most common types of bacteria found in the vaginal microbiota in healthy women [15]. Five community state types (CSTs) have been identified globally. Four of these types (I, II, III, and V) mainly consist of one or more species of *Lactobacillus* [16]. The dominance of lactobacilli in the vaginal microbiota is related to estrogen production and the accumulation of glycogen in the top layers of vaginal epithelial cells [17]. An imbalance in the vaginal microbiota, such as bacterial vaginosis (BV), could render women more susceptible to high-risk HPV infection and lower the immune system’s ability to clear the virus [18]. The main way that the microbiota protects the female reproductive tract is believed to be through the production of lactic acid by *Lactobacillus* species. These bacteria use anaerobic metabolism to break down glycogen from vaginal mucosal cells, producing lactic acid, which helps to maintain vaginal health [19]. Other researchers have studied how vaginal lactobacilli can kill cervical tumor cells using laboratory (in vitro) experiments [20]. Many studies have examined the relationship between the vaginal microbiota, HPV infection, and cervical disease. They have found that changes in the microbiota, especially reduced mucus production and less effective virus trapping, may cause women to acquire HPV infections more easily, reactivate dormant infections, or cause delays in clearing the virus [6,21]. Motevaseli et al. found that women affected by HPV had significantly lower levels of *Lactobacillus crispatus* [21,22].

We hypothesized that cervical lesion progression stages, rather than HPV status alone, are the primary drivers of distinct microbiome shifts, influencing microbial diversity, the community composition, and the predicted functional potential. Thus, we aimed to characterize, for the first time in Morocco, the cervico-vaginal microbiota in healthy women, HPV-positive women with pre-cancerous lesions, and post-treatment cervical cancer patients.

## 2. Materials and Methods

### 2.1. Sample Collection and Study Design

The study included a total of 247 women, divided into three clinical groups and one control group: (I) 104 women diagnosed with CC and treated with radiochemotherapy; (II) 43 women with pre-cancerous cervical lesions, all of whom tested positive for high-risk HPV; and (III) 100 healthy control women, all negative for HPV infection. All cervical disease cases were histologically confirmed as squamous cell carcinoma. No cases of adenocarcinoma or adenosquamous carcinoma were observed. Among the 104 women in the post-treatment group, HPV testing revealed a detection rate of 33.81%, with high-risk HPV genotypes accounting for 70.21% of positive cases. In contrast, only 8% of the control women tested positive for HPV. Eight different HPV genotypes were identified, with HPV16 being the most common among both cases (59.57%) and controls (75%), followed by HPV53 and HPV18. High-risk HPV types included HPV16, HPV18, HPV31, and HPV66, while low-risk types included HPV53, HPV83, HPV89, and HPV62. Some genotypes were unidentifiable using the NCBI reference database.

The 247 participants were divided into three main study groups based on their clinical condition and HPV status: (I) CC cases post-radiochemotherapy with known HPV status; (II) pre-cancerous cases with confirmed high-risk HPV infection; and (III) HPV-negative control women. Persistent HPV infection was defined as the continued presence of the same HPV genotype for more than 12 months, while transient infection referred to cases where the virus was cleared within one year.

This research was conducted between November 2020 and April 2023 in two Moroccan cities, Rabat and Casablanca. CC cases were recruited from the National Institute of Oncology in Rabat, and control participants were enrolled from the Cheikh Khalifa Ibn Zaid Hospital in Casablanca. The study protocol was approved by the Ethics Committee for Biomedical Research, Faculty of Medicine and Pharmacy of Rabat, Mohammed V University, Morocco (approval code: 21/20, approval date: 20 February 2020). Written informed consent was obtained from all participants prior to enrollment.

### 2.2. Bacterial DNA Extraction and Polymerase Chain Reaction (PCR) Quantification

Total genomic DNA was extracted from cervical swab samples collected from 247 women using the phenol/chloroform extraction method. Briefly, samples were lysed and treated with proteinase K and SDS, followed by sequential extraction with phenol, phenol/chloroform/isoamyl alcohol (25:24:1), and chloroform. DNA was precipitated with cold ethanol and sodium acetate, washed with 70% ethanol, and resuspended in TE buffer. DNA quality and quantity were assessed using agarose gel electrophoresis and spectrophotometric measurements on a BioSpectrophotometer (Eppendorf, Hamburg, Germany).

The V5–V6 hypervariable regions of the bacterial 16S rRNA gene were assessed using a primer pair that included custom sequence (CS) adapters at the 5′ end: CS1-719F/ACACTGACGACATGGTTCTACA-AACMGGATTAGATACCCKG and CS2-1115R TACGGTAGCAGAGACTTGGTCT-AGGGTTGCGCTCGTTG [23]. Each 25 μL PCR reaction contained 1× Platinum Direct PCR Universal Master Mix (ThermoFisher, Rabat, Morocco), 0.2 μM of each primer, and approximately 10 ng of template DNA. Amplification was performed in duplicate using a Mastercycler X50s (Eppendorf, Hamburg, Germany) with the following conditions: initial denaturation at 94 °C for 3 min, followed by 35 cycles of 94 °C for 30 s, 55 °C for 30 s, and 72 °C for 1 min, with a final extension at 72 °C for 5 min. Negative controls (sterile Milli-Q water; MilliporeSigma, Burlington, MA, USA) and positive controls were included in each PCR run.

### 2.3. Library Preparation and Sequencing

Preparation of the bacterial 16S rRNA gene amplicon libraries was carried out as previously described by Legeay et al. [24]. In brief, initial PCR products were purified using Agencourt AMPure XP magnetic beads (Beckman Coulter, Brea, CA, USA) and subsequently eluted in 10 mM Tris buffer (pH 8.5). A second round of PCR was then performed to incorporate Illumina sequencing adapters and unique index barcodes. Each 50 μL indexing reaction consisted of 5 μL of purified PCR product, 2.5 μL of Fluidigm Access Array Barcode 384, and 1× KAPA HiFi HotStart ReadyMix (Roche Sequencing Solutions, Seattle, WA, USA). The thermocycling conditions were as follows: initial denaturation at 95 °C for 3 min; 8 cycles of 95 °C for 30 s, 55 °C for 30 s, and 72 °C for 30 s; and a final extension at 72 °C for 5 min. Indexed amplicons were again purified using Agencourt AMPure XP (Beckman Coulter, Brea, CA, USA), and DNA concentrations were quantified using a Qubit Fluorometer with the DNA High Sensitivity (HS) assay kit (ThermoFisher, Témara, Morocco). Final library quantification, normalization, and pooling were performed according to the manufacturer’s recommendations (Illumina). Sequencing of the 16S rRNA gene libraries was conducted on an Illumina MiSeq platform (Illumina, Paris, France) using a MiSeq Reagent Kit v3, configured for 300-cycle paired-end reads.

### 2.4. Bioinformatic Analysis

Raw sequencing data were processed using the DADA2 pipeline implemented in R version 4.3.3 [25]. Quality filtering was applied to exclude reads with Phred scores below 30. Following the removal of primers and adapter sequences, forward reads were filtered and denoised using DADA2’s error correction model. Reads exceeding 10 base pairs in length were subsequently clustered into amplicon sequence variants (ASVs). The taxonomic assignment of ASVs was performed using the most recent SILVA reference database [26]. The raw reads were deposited into the Sequence Read Archive (NCBI accession number: PRJNA1262983).

### 2.5. Statistical Analysis

To account for differences in sequencing depth between samples, the abundance of ASVs was converted into relative proportions using the “compositional” option in the phyloseq package in R. Bacterial alpha diversity was measured using the Shannon and Simpson indices at the ASV level, also with phyloseq. Beta diversity was analyzed using the Bray–Curtis and UniFrac distance measures to compare microbial communities between samples. Statistical testing of beta diversity was performed with PERMANOVA. Differences in data spread between groups were tested using the betadisper function.

To identify key microbial features, random forest models were built using the randomForest package in R. The most accurate models were used to identify the microbial taxa that best distinguished between sample groups. Significant associations between taxa and sample conditions were identified using indicspecies.

## 3. Results

### 3.1. Demographic and Clinical Characteristics

A total of 243 women participated in the study, of which 104 (42.8%) were treated CC cases, 100 (41.2%) were controls, and 39 (16.1%) had high-grade pre-cancerous lesions. Cases were older, with 60.9% aged > 56 years, versus 48.0% of controls aged < 41 years, and were less often married (58.5% vs. 78.0%), insured (84.8% vs. 99%), or urban/suburban residents (64.6% vs. 90.0%). Educational attainment differed, with 73.8% of cases being illiterate, versus 65% of controls having higher education. Reproductive history showed menarche ≤ 12 years in 23.8% of cases versus 51% of controls, post-menopause in 90.2% versus 35%, and early sexual debut (<18 years) in 74.2% versus 25.8%. Sexual health indicators likewise varied: partner monogamy was noted in 52.6% of cases versus 92.7% of controls, prior STIs in 40.2% versus 25%, and smoking in 36.6% versus 15%. Among cases, the FIGO stage was II in 53.3%, III in 23.8%, I in 16.6%, and unclassified in 7.1%. High-risk HPV genotyping identified HPV16 in 59.6%, HPV53 in 14.8%, HPV18 in 6.4%, and HPV83, -31, -89, -66, and -62 in 2.1% each; all 39 pre-cancerous women tested positive for HPV16/18 (Table 1).

Unfortunately, complete metadata for the group with pre-cancerous lesions were not consistently available due to sampling and clinical reporting limitations during participant recruitment.

To assess the independent associations between clinical group status and participants’ characteristics, we conducted a multiple logistic regression analysis. Variables included in the model were age, marital status, education level, smoking status, menopausal status, sexual debut, STI history, and residence type. The results are presented in Supplementary Table S1, and early sexual debut, smoking, and post-menopausal status were the strongest predictors of being in the cancer or pre-cancer group compared to healthy controls.

### 3.2. Microbial Diversity and Taxonomic Composition

**Figure 1:**
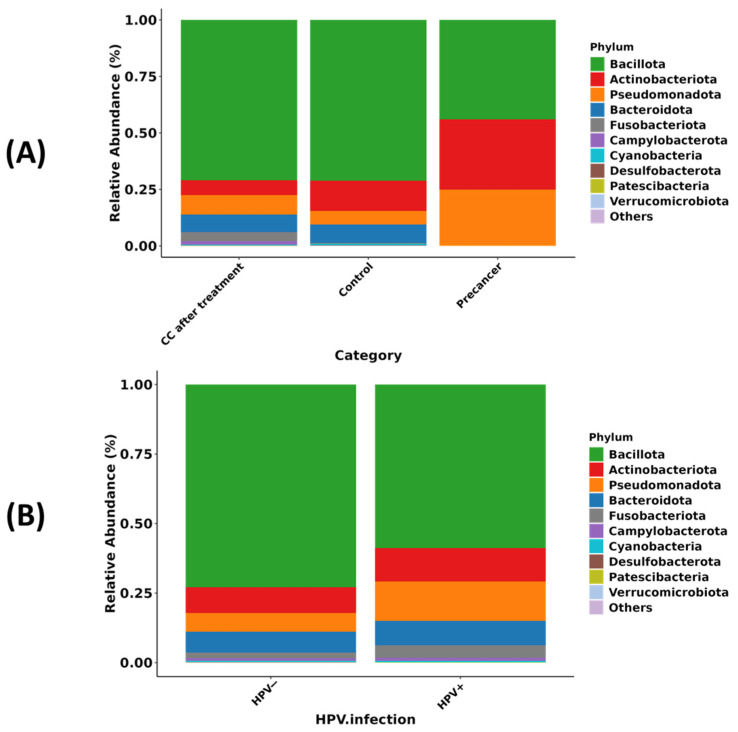
Phylum-level composition of the cervico-vaginal microbiome in Moroccan women. (**A**) Relative abundance of the ten dominant bacterial phyla in CC cases after radiochemotherapy (CC after treatment), healthy controls, and high-grade pre-cancerous lesion groups. (**B**) Relative abundance of the same phyla stratified by HPV infection status (HPV^−^ versus HPV^+^).

In healthy controls, Bacillota—primarily *Lactobacillus*—accounts for approximately 70% of the bacterial community and remains at about 70% in women after CC treatment, whereas, in the pre-cancer group, Bacillota declines to roughly 45%, with Pseudomonadota rising from about 6% to 25% and Actinobacteriota from about 12% to 30%; Fusobacteriota and Bacteroidota each stay below 5%, with minimal change. A similar pattern regarding HPV status shows Bacillota decreasing from approximately 73% in HPV-negative women to about 60% in HPV-positive women, while Pseudomonadota increases to 15%, Bacteroidota to 10%, Actinobacteriota to 10%, and Fusobacteriota to 5% (Figure 1A,B).

#### 3.2.1. Alpha Diversity

**Figure 2:**
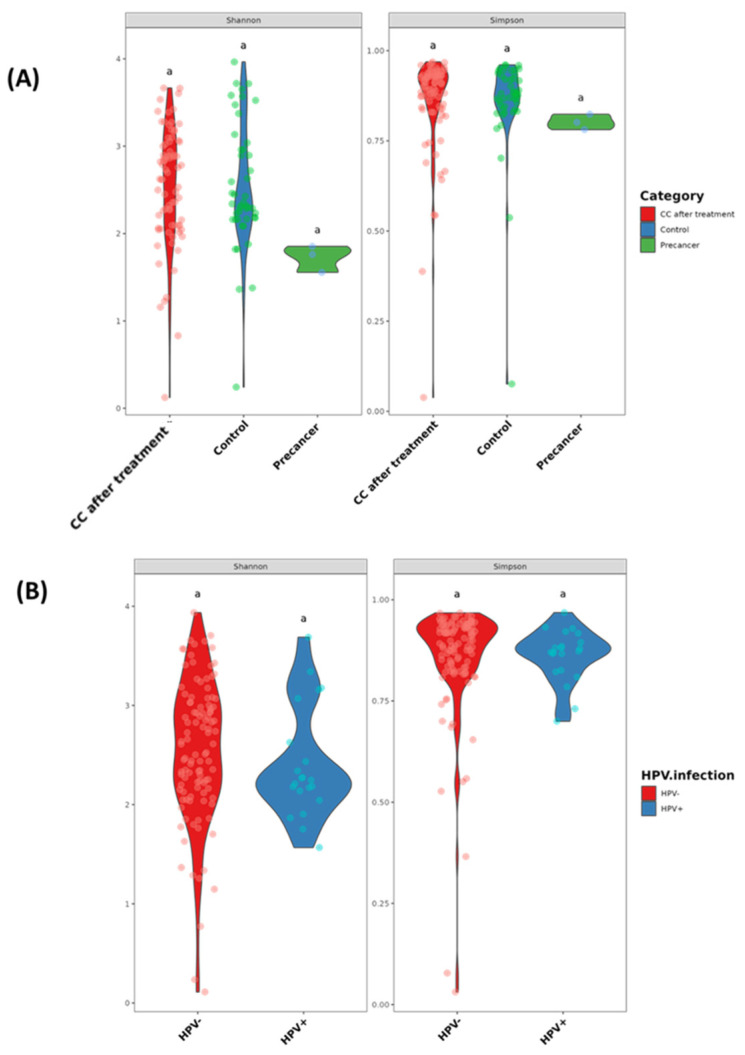
Alpha diversity of the cervico-vaginal microbiome in Moroccan women. (**A**) Shannon and Simpson diversity indices in CC cases after radiochemotherapy (CC after treatment), healthy controls, and high-grade pre-cancerous lesion groups. (**B**) Shannon and Simpson diversity indices stratified by HPV infection status. Groups sharing the same letter (e.g., “a”) are not significantly different (*p* > 0.05).

The Shannon diversity (richness and evenness) and Simpson diversity (dominance) indices do not differ significantly across clinical stages or HPV groups (all pairwise comparisons carry the same significance letter “a”, indicating *p* > 0.05). The median Shannon values are approximately 2.5 for controls, 1.8 for pre-cancer, and 2.5 for post-treatment, while the Simpson medians cluster near 0.90, 0.80, and 0.90, respectively. When stratified by HPV status, HPV-negative samples have a median Shannon index of 2.7 and Simpson index of 0.91, and HPV-positive samples have values of 2.3 and 0.86, demonstrating that neither the disease stage nor HPV carriage significantly alters the within-sample diversity (Figure 2A,B).

#### 3.2.2. Beta Diversity

**Figure 3:**
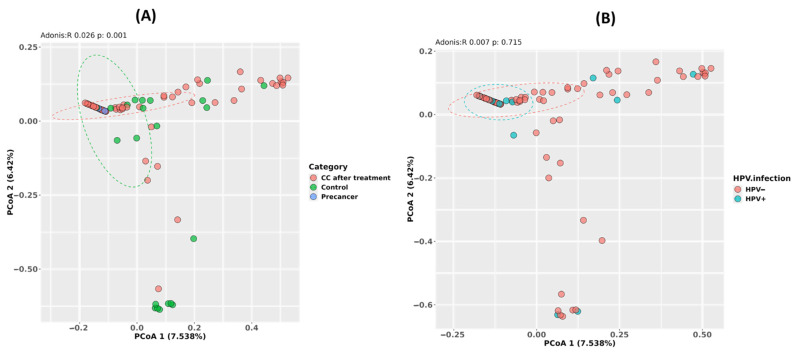
Beta diversity of the cervico-vaginal microbiome in Moroccan women. (**A**) Principal-coordinate analysis (PCoA) based on Bray–Curtis distances for CC cases after radiochemotherapy (CC after treatment), healthy controls, and high-grade pre-cancerous lesion groups (Adonis R = 0.296, *p* = 0.001). (**B**) PCoA based on the same distances, stratified by HPV infection status (HPV^−^ versus HPV^+^; Adonis R = 0.087, *p* = 0.713).

The Bray–Curtis principal coordinate analysis (PCoA) of the community structure reveals a clear shift by clinical stage but not by HPV status. The first two axes explain 7.5% (PCoA1) and 6.4% (PCoA2) of the total compositional variance. Samples from the CC post-treatment group cluster distinctly to the right along PCoA1, separated from control and pre-cancer samples near the origin, and the PERMANOVA confirms that the clinical stage accounts for 2.6% of the variance (Adonis R = 0.026, *p* = 0.001). In contrast, HPV-negative and HPV-positive samples overlap broadly on the same PCoA plot, with the PERMANOVA showing a non-significant effect of HPV status (Adonis R = 0.007, *p* = 0.715), indicating that viral carriage does not drive large-scale community restructuring (Figure 3A,B).

#### 3.2.3. Phylum-Level Microbiome Shifts Across Cervical Disease Stages and Post-Treatment

**Figure 4:**
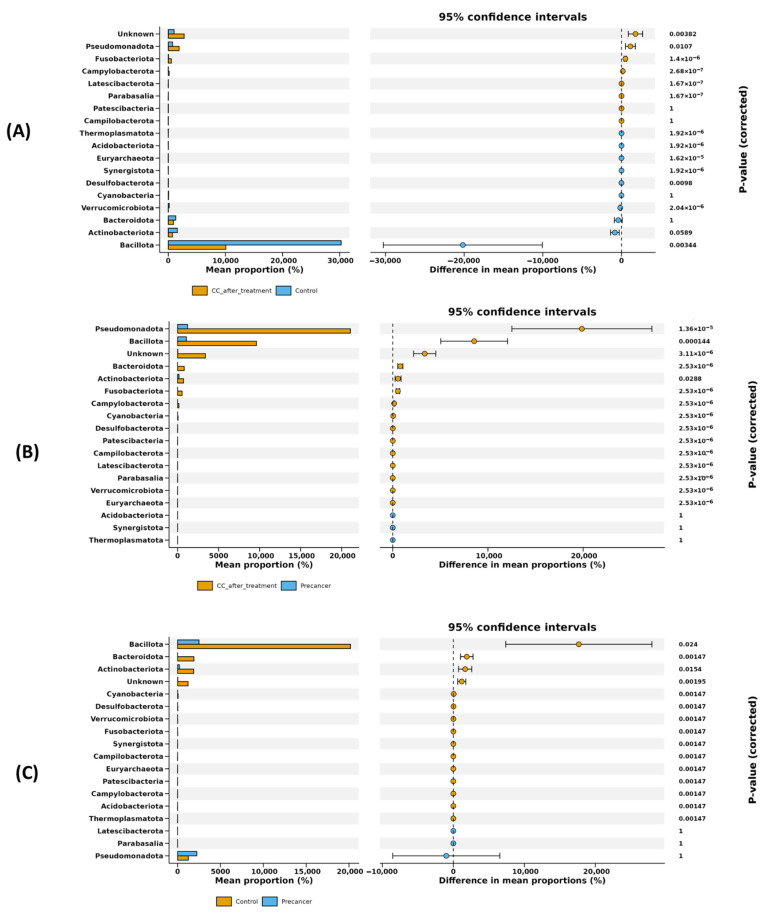
Statistical analysis of differential phylum-level abundances among cervical scraping sample groups. Differential phyla were filtered according to the difference in mean proportion with 95% confidence intervals. (**A**) Differential phyla in the comparison of post-treatment CC cases versus healthy controls. (**B**) Differential phyla in the comparison of post-treatment CC cases versus high-grade pre-cancerous lesion samples. (**C**) Differential phyla in the comparison of high-grade pre-cancerous lesion samples versus healthy controls.

Differential abundance analysis at the phylum level (S1) and complementary mean difference plotting with 95% confidence intervals (Figure 4) together reveal that Bacillota, Verrucomicrobiota, Synergistota, Campylobacterota, Fusobacteriota, Pseudomonadota, Latescibacterota, Thermoplasmatota, and Desulfobacterota showed major shifts across CC progression and post-treatment (S1; Figure 4). After radiochemotherapy, Bacillota showed a marked fivefold decrease (adjusted *p*-value = 0.003; *p* < 1 × 10^−15^), with its mean relative abundance decreasing by approximately 20%. Verrucomicrobiota and Synergistota also decreased, while Campylobacterota showed a strong increase, with a log fold change of approximately +6 (*p* < 1 × 10^−21^). Other low-abundance phyla, including Fusobacteriota, Pseudomonadota, Latescibacterota, Thermoplasmatota, and Desulfobacterota, were enriched significantly (adjusted *p*-values < 0.05). These differences were confirmed by the mean difference plots, with the 95% confidence intervals excluding zero (Figure 4A). In comparison with high-grade pre-cancerous lesions, Bacillota increased significantly (adjusted *p*-value = 1.4 × 10^−4^), and Pseudomonadota showed a further increase (adjusted *p*-value = 1.3 × 10^−5^). Smaller increases were observed in Actinobacteriota and Bacteroidota (adjusted *p*-values ≤ 0.03), all supported by the 95% confidence intervals in Figure 4B. During early lesion development, Bacillota was significantly reduced (adjusted *p*-value = 0.024), while Bacteroidota and Actinobacteriota increased (adjusted *p*-values ≤ 0.016). Cyanobacteria, Desulfobacterota, and Verrucomicrobiota also showed significant enrichment (adjusted *p*-value = 0.001). These trends are supported by the mean difference analysis shown in Figure 4C.

#### 3.2.4. Clinical Stage-Driven Cervico-Vaginal Microbiome Reconfiguration Exceeds HPV-Associated Shifts

**Figure 5:**
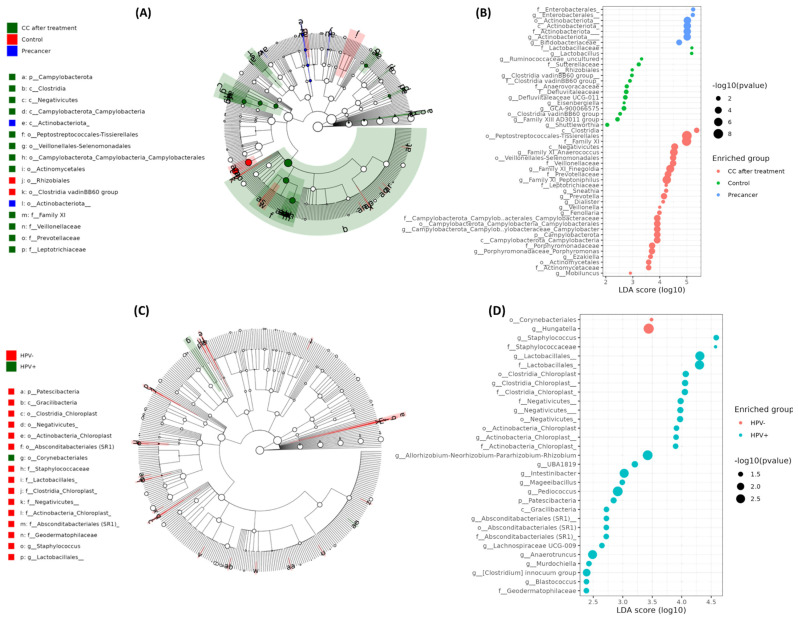
LEfSe-derived biomarkers at the phylum and genus levels in cervical scraping samples. (**A**) Cladogram depicting the taxonomic hierarchy of phylotype biomarkers among clinical groups (CC after treatment in red, healthy controls in green, pre-cancer in blue). Each filled circle denotes one biomarker; the circle diameter is proportional to its LDA effect size. Phylum and class names are shown on the tree; orders, families, and genera are keyed alongside. (**B**) LDA scores ranking the same biomarkers by effect size for the clinical comparison in (**A**). Larger bars indicate more discriminative phylotypes. (**C**) Cladogram of phylotype biomarkers distinguishing HPV^−^ (green) from HPV^+^ (red) women. Circle size again reflects LDA effect size, with higher-level taxa labeled on the tree and lower-level taxa in the legend. (**D**) LDA score plot listing genus- and family-level biomarkers enriched in HPV^−^ versus HPV^+^ groups, ordered by descending effect size (α < 0.05).

Dot plots and LEfSe analysis revealed a three-phase microbial shift during disease progression, largely independent of HPV infection. In healthy controls, the cervico-vaginal microbiota was dominated by *Lactobacillus* and Lactobacillaceae, with average read counts of 5 × 10^5^ and LDA scores of 4.2, followed by *Bifidobacterium* and Ruminococcaceae (LDA: 3.7–3.9). In pre-cancerous lesions, the *Lactobacillus* levels decreased to less than 2 × 10^5^ reads, while Enterobacterales increased to 8 × 10^5^ reads (LDA: 4.1), along with the enrichment of Staphylococcales and actinobacterial orders (LDA: 3.4–3.8). Post-treatment samples showed the suppression of Enterobacterales, with the increased abundance of strict anaerobes such as Peptostreptococcales-Tissierellales, Family XI genera (*Peptoniphilus*, *Finegoldia*), Clostridia, and *Porphyromonas*, with read counts between 2 and 3 × 10^5^ and LDA scores ranging from 3.2 to 4.0. *Lactobacillus* partially recovered to 1.3 × 10^5^ reads (Figure 5A–D). HPV stratification showed only modest microbial changes, with higher abundances of chloroplast-related Actinobacteria, Negativicutes, and gut-associated anaerobes such as Intestinibacter and Mageeibacillus in HPV-positive samples (LDA: 3.0–3.6) and slight increases in Corynebacteriales and Hungatella in HPV-negative samples (LDA: 3.1).

**Figure 6:**
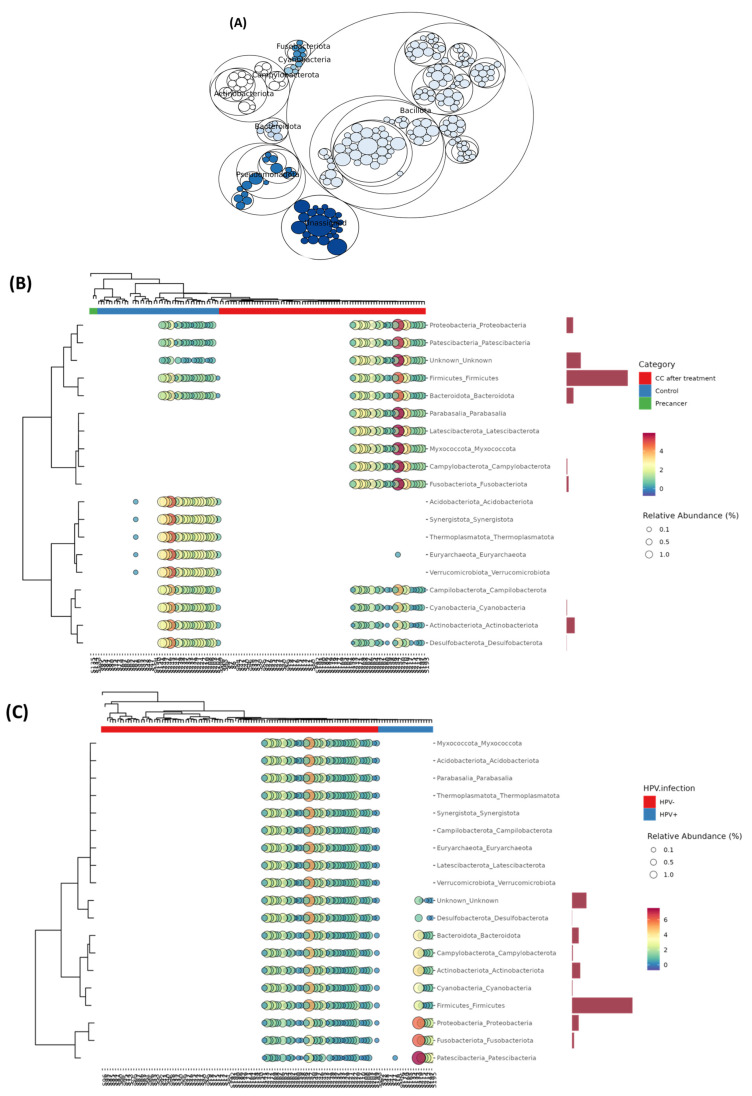
Co-occurrence network and hierarchical clustering of the cervico-vaginal microbiome in Moroccan women. (**A**) Network representation of phylum-level co-occurrence patterns, generated using SparCC correlations. Each node denotes a bacterial phylum and edges indicate significant and positive associations (r ≥ 0.5, *p* < 0.05). (**B**) Heat tree and bubble plot of genus-level relative abundances clustered by clinical group (red = CC after treatment; green = control; blue = pre-cancer), with bubble size proportional to abundance and side bars showing mean differences between CC post-treatment and controls. (**C**) Heat tree and bubble plot of genus-level relative abundances clustered by HPV infection status (red = HPV^+^; teal = HPV^−^).

The maptree constructed from the top 200 OTUs confirmed a Firmicutes core formed by large Bacillota clusters, surrounded by substantial Pseudomonadota and Actinobacteriota aggregates. Peripheral expansions of Campylobacterota, Fusobacteriota, Cyanobacteria, and Bacteroidota were observed, alongside an “Unassigned” cluster representing unresolved taxonomic diversity (Figure 6A).

Phylum-level bubble heatmaps (clinical staging) indicated that control samples contained 70% Firmicutes, 6% Proteobacteria, and 12% Actinobacteriota. Pre-cancerous lesions were marked by a drop in Firmicutes to 45%, with an increase in Proteobacteria to 25% and Actinobacteriota to 30%, while Fusobacteriota and Campylobacterota remained below 4%. Post-treatment samples displayed 50% Firmicutes, 12% Proteobacteria, 20% Peptostreptococcales-Family XI, and 2% Patescibacteria. The HPV status influenced the phylum proportions only slightly, with differences of one to four percentage points (Figure 6B,C).

Co-occurrence networks (Figure S2A–E) revealed progressive shifts in central microbial hubs across clinical conditions. In healthy controls, Akkermansiaceae was the dominant hub, positively associated with Lactobacillaceae, Rikenellaceae, Enterobacteriaceae, and Eggerthellaceae and negatively associated with Ruminococcaceae and Lachnospiraceae. In HPV-positive samples, Neisseriaceae took the central position, correlating positively with Sutterellaceae, Moraxellaceae, Staphylococcaceae, and Family XI and negatively with Bacteroidaceae, Lachnospiraceae, and Veillonellaceae. In HPV-negative samples, Lactobacillaceae remained central. In pre-cancer lesions, Butyricicoccaceae emerged as the dominant hub, with positive links to Family XI, Enterobacteriaceae, Coriobacteriaceae, and Eggerthellaceae and negative links to Bacteroidaceae and Lachnospiraceae. In post-treatment samples, Pasteurellaceae became central, positively associated with Family XI, Prevotellaceae, and other Firmicutes families and negatively with Bacteroidaceae and Lachnospiraceae.

### 3.3. Stepwise Shifts in Predicted Metabolic Functions Across Clinical Stages

**Figure 7:**
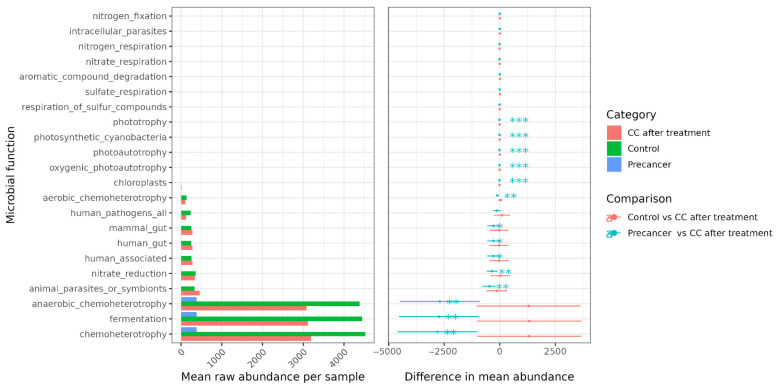
FAPROTAX predicted functional group profiling of the cervico-vaginal microbiome. * *p* < 0.05, ** *p* < 0.01, *** *p* < 0.001.

FAPROTAX functional predictions revealed a progressive decline in classical heterotrophic functions from control samples to post-treatment and pre-cancer groups. Chemo-heterotrophic activity decreased from 4700 reads per sample in controls to 3400 in CC post-treatment and 2600 in the pre-cancer group. Anaerobic chemo-heterotrophy followed a similar trend, dropping from 4800 to 2900 and 2300 reads, respectively. Fermentative function also declined from 4400 in controls to 3200 post-treatment and 2150 in pre-cancer lesions. Host-adapted metabolic pathways showed parallel reductions. The “human_gut” category declined from 220 reads in controls to 120 in post-treatment samples and less than 80 in the pre-cancer group. “Mammal_gut” decreased from 180 to 110 and less than 80, respectively. Reads associated with the “human_associated” function dropped from 450 to 300 and 200, while “animal_parasites_or_symbionts” fell from 300 to 200 and 120. The “nitrate_reduction” function decreased from 330 to 180 and 120 reads and “human_pathogens_all” from 180 to 90 and 60. Aerobic chemo-heterotrophy dropped from 150 to 80 and 50. Chloroplast-related functions remained consistently low, with values ≤ 6 across all groups. In contrast, specialist metabolic functions increased markedly in pre-cancer samples. Nitrogen fixation increased from two reads in controls and four in CC to 25 in pre-cancer. Nitrate respiration increased from values ≤ 6 in controls to 35 reads in pre-cancer. Sulfate respiration rose from similar low baseline levels to 20 reads. Functions related to the respiration of sulfur compounds increased to 22 reads, phototrophy to 12, oxygenic photoautotrophy to 15, and general photoautotrophy to 12. Photosynthetic cyanobacteria increased to 10 reads, nitrogen respiration to five, aromatic compound degradation to 15, and intracellular parasites to two reads. Each shift is highly significant (*p* < 0.001), underscoring a metabolic drift in pre-cancer lesions toward taxa exploiting inorganic electron acceptors and light (Figure 7).

### 3.4. Host and Behavioral Factors Drive Cervico-Vaginal Microbiome Variation

**Figure 8:**
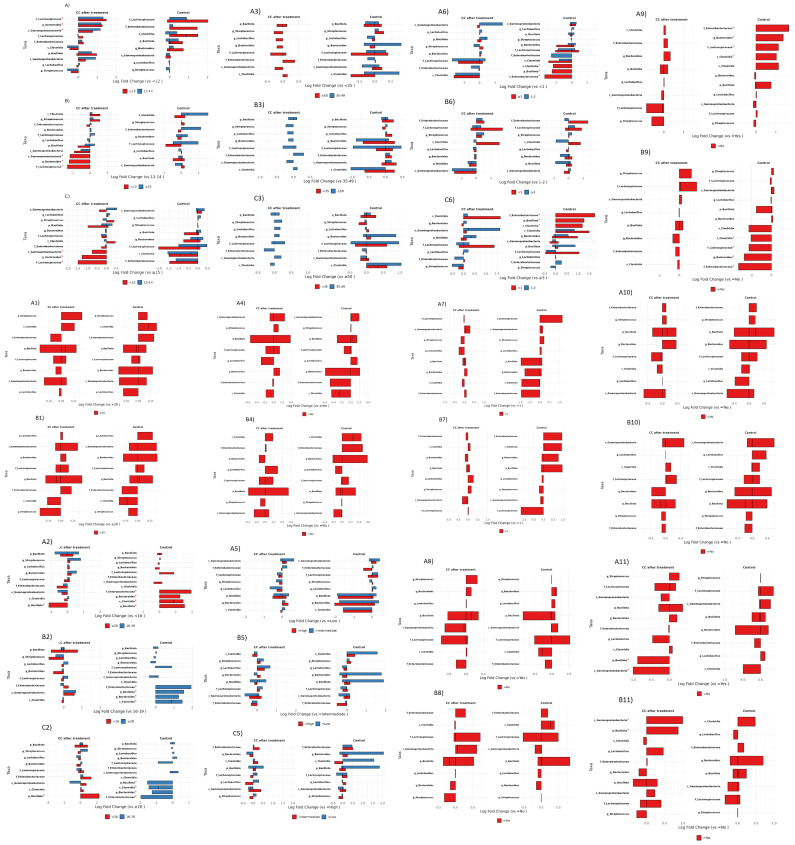
Differential microbiota analysis of cervico-vaginal samples according to host and behavioral factors. (**A**–**C**) Comparison according to age at first menstruation. (**A1,B1**) Comparison according to age at first pregnancy. (**A2**–**C2**) Comparison according to age at first intercourse. (**A3**–**C3**) Comparison according to age group. (**A4,B4**) Comparison according dietary habits. (**A5**–**C5**) Comparison according to menopause. (**A6**–**C6**) Comparison according to number of pregnancies. (**A7**,**B7**) Comparison according to number of sexual partners of husband. (**A8**,**B8**) Comparison according to use of contraceptives. (**A9**,**B9**) Comparison according to sexually transmitted infections. (**A10**,**B10**) Comparison according to tobacco exposure. (**A11**,**B11**) Comparison according to use of condoms. * Indicates taxa with statistically significant differences in relative abundance between clinical groups (adjusted *p* < 0.05).

Figure 8 shows that age at menarche, age at first pregnancy, chronological age, sexual debut, reproductive history, and sexual behaviors each exert distinct and sometimes clinical state-specific effects on the cervical microbiota.

Age at first menstruation demonstrated the strongest and most consistent associations with the cervico-vaginal microbiota. In CC post-treatment samples, late menarche (≥15 years) was associated with the enrichment of Lachnospiraceae (log-fold change 1.2), Bacteroides (0.8), and Gammaproteobacteria (0.7). In healthy controls with late menarche, the increases were more pronounced for Lachnospiraceae (1.9) and Enterobacteriaceae (1.1).

Comparisons between intermediate (12–14 years) and early menarche (<12 years) revealed contrasting patterns: in CC post-treatment samples, Gammaproteobacteria, Bacteroides, and Lachnospiraceae decreased by 1.2, 1.1, and 0.9 log-fold, respectively, whereas, in controls, Clostridia and Lachnospiraceae increased by 1.1 and 1.0. Direct comparisons of early versus late menarche further confirmed these trends, showing the depletion of Gammaproteobacteria (−1.7), Bacteroides (−1.3), and Lachnospiraceae (−1.4) in CC and the enrichment of Lachnospiraceae (1.5), Clostridia (1.1), and Enterobacteriaceae (1.0) in controls (all *p* < 0.05).

Other significant associations were observed with reproductive history and behaviors. Controls with three or more pregnancies exhibited higher levels of Enterobacteriaceae, Bacillota, and Clostridia (*p* < 0.05). Similarly, a history of sexually transmitted infections (STIs) in controls correlated with increased abundances of Enterobacteriaceae, Bacteroides, Lachnospiraceae, and Clostridia (*p* < 0.05). Among CC post-treatment samples, contraceptive users showed elevated Gammaproteobacteria and Bacillota (*p* < 0.05). Moderate shifts, although not quantified, were observed in relation to sexual debut, age at first pregnancy, and chronological age. For example, CC post-treatment participants with a sexual debut at or after 20 years had increased Bacillota (0.8), while controls with a sexual debut before 16 years showed elevated Enterobacteriaceae (1.5), Bacteroides (1.0), Clostridia (1.0), and Bacillota (2.0). Regarding age at first pregnancy, CC participants aged 20 years or older had higher Streptococcus, Gammaproteobacteria, and Bacillota but lower *Lactobacillus*, Bacteroides, Lachnospiraceae, Enterobacteriaceae, and Clostridia. In contrast, controls of the same age group displayed increased Bacteroides, Gammaproteobacteria, and Bacillota, with decreases in Streptococcus, Clostridia, Enterobacteriaceae, Lachnospiraceae, and *Lactobacillus*.

Chronological age also influenced the microbiota composition. In CC samples, women aged 50 years or older, compared to those under 35, showed modest increases in Enterobacteriaceae and Lachnospiraceae (0.3). Controls aged 35–49 versus under 35 had elevated Bacteroides (0.5) and Gammaproteobacteria (0.3), while controls under 35 versus 35–49 years showed higher Lachnospiraceae (0.6). Additionally, controls aged 50 or older versus 35–49 exhibited increases in Bacillota (0.2), with the 35–49 group enriched in Clostridia (0.7) and Lachnospiraceae (0.6).

Finally, factors such as diet, menopausal status, intercourse during menstruation, and tobacco exposure resulted in only minor, non-significant variations across key taxa including Bacillota, Gammaproteobacteria, Bacteroides, Lachnospiraceae, Clostridia, Streptococcus, and Enterobacteriaceae in both the CC and control groups (Figure 8).

## 4. Discussion

The cervico-vaginal region is protected by three key defenses: the intact structure of the vulva and vaginal walls, an acid-producing *Lactobacillus*-rich microbiota, and the cervical mucus plug that blocks pathogens from moving upward. The disruption of any of these defenses predisposes individuals to HPV acquisition, persistence, and progression to cervical pre-cancer and cancer [16,27].

In our cohort of 243 women, we observed that treated CC cases were older, less often married or insured, and had an earlier menarche and sexual debut than controls—factors that have previously been linked to both HPV persistence and vaginal dysbiosis [23,24]. The predominance of high-risk HPV genotypes (HPV16 in 59.6% of cases) underscores the etiologic role of these strains in lesion progression [2,23].

In a Nigerian screening cohort of 278 women, Dareng et al. [28] found that 23.7% harbored prevalent high-risk human papillomavirus (hrHPV); HIV-negative hrHPV^+^ women exhibited significantly reduced *Lactobacillus* and enriched anaerobes *Prevotella* and Leptotrichia according to LEfSe analysis (LDA > 2.0, *p* < 0.05). They identified four community state types (CSTs) (I, III, IV-B, and VI), with CST IV-B (low *Lactobacillus*, high *Gardnerella*/anaerobes) being the most prevalent (50%), regardless of HPV or HIV status, and CSTs I/III were inversely associated with hrHPV (albeit not reaching significance after multivariable adjustment). Importantly, in HIV-negative women only, the weighted UniFrac distances differed by hrHPV status (*p* = 0.02), and LEfSe pinpointed Prevotellaceae and Leptotrichiaceae as biomarkers of hrHPV infection [25]. Our Moroccan cohort similarly showed that hrHPV presence alone exerted modest shifts—Bacillota falling from 73% to 60% and Pseudomonadota rising to 15%—while the clinical stage drove far larger community reorganization. The congruence between these geographically distinct populations underscores that hrHPV infection correlates with the depletion of protective lactobacilli and modest anaerobe blooms, but that the onset of histologic lesions precipitates more profound dysbiosis. Beyond HPV status, clinical staging dictated major shifts. Bacillota fell from 70% in controls to 45% in pre-cancer cases, with Pseudomonadota and Actinobacteriota surging to 25% and 30%, respectively, paralleling the CST IV signature in high-grade CIN [26]. Post-treatment communities partially recovered *Lactobacillus* (50%) yet remained enriched in strict anaerobes (Peptostreptococcales, *Porphyromonas*), indicating that conventional therapy does not fully restore eubiotic CSTs. The congruence between these geographically distinct populations underscores that, while HPV infection correlates with the depletion of protective Lactobacillus and modest anaerobe blooms, it is the histologic lesion stage that precipitates deeper vaginal dysbiosis.

Alpha diversity (Shannon: 2.5 controls, 1.8 pre-cancer, 2.5 post-treatment; Simpson: 0.90, 0.80, 0.90) did not differ significantly by stage or HPV status in Morocco. This stability is echoed in U.S. [29] and Chinese [30] cohorts, where richness and evenness remain unchanged despite compositional shifts. In contrast, beta diversity (Bray–Curtis PCoA) clearly separated clinical stages (Adonis R = 0.026, *p* = 0.001) but not HPV carriage (R = 0.007, *p* = 0.715)—a pattern also observed in Nigeria [25]—indicating that the lesion stage, rather than viral status alone, drives the overall community structure.

The phylum-level volcano analyses in our study highlighted a 20% Bacillota depletion post-treatment (adj. *p* = 0.003; logFC= −5) and blooms of Campylobacterota and Fusobacteriota (logFC= +6 to +21). Similar metagenomic enrichments of Campylobacter and *Fusobacterium* have been reported in Chinese CC patients [30] and in Tanzanian high-grade lesions [31].

Functionally, FAPROTAX showed a gradual decrease in chemo-heterotrophy, with predicted reads falling from 4700 in healthy controls to 2600 in the pre-cancer group. Similar declines were observed in fermentation and in pathways linked to the human gut, where functional reads dropped from 220 to fewer than 80. At the same time, the pre-cancer group showed increased levels of functions such as nitrogen fixation, nitrate and sulfate respiration, and phototrophy. All differences were statistically significant (*p* < 0.001). This metabolic reprogramming parallels Chinese PICRUSt-KEGG findings of depressed carbohydrate metabolism and enriched xenobiotic degradation pathways in CIN [30] and U.S. shotgun-metagenomic reports of increased peptidoglycan synthesis in cancer [28]. Although partial Lactobacillus recovery was observed in the post-treatment group, the potential influence of chemoradiotherapy on the cervico-vaginal microbiome must also be considered. Although the partial restoration of Lactobacillus dominance was observed in post-treatment patients, the persistent enrichment of anaerobes such as Porphyromonas and Peptostreptococcus, along with altered predicted functional pathways, suggests that treatment may lead to long-lasting ecological shifts. A clinical study of pelvic intensity-modulated radiotherapy (IMRT) with concurrent chemotherapy in cervical cancer patients demonstrated significant alterations in vaginal microbial composition over time, including increased abundances of Gammaproteobacteria, Prevotella, and other environmental taxa [32]. A recent review reported treatment-related reductions in microbial diversity and community stability in gynecologic cancer patients, with lasting disturbances in the mucosal microbiota beyond the treatment period [33]. These findings highlight the need for future longitudinal studies to assess the microbiome recovery dynamics following cancer therapy. Taken together, our results underscore that the microbial composition is not only influenced by the disease stage but may also be modulated by clinical interventions such as chemoradiotherapy. Our findings suggest that treatment-associated microbiome changes could impact vaginal homeostasis and warrant closer post-treatment clinical surveillance.

Late menarche (≥15 years) correlated with Lachnospiraceae and Bacteroides enrichment in both Moroccan CC post-treatment and controls (logFC= 1.2–1.9), echoing Korean twin-study results linking delayed menarche to non-*Lactobacillus* CSTs [34]. In Nigeria, early sexual debut (<16 years) was associated with higher *Prevotella* and Bacteroides in hrHPV^+^ women [25]. Controls with ≥ 3 pregnancies or an STI history showed elevated Enterobacteriaceae and Clostridia in Morocco, paralleling meta-analyses from diverse settings [24]. Contraceptive use in Moroccan CC post-treatment enriched Gammaproteobacteria and Bacillota (*p* < 0.05), as reported in South African and U.S. cohorts [35].

One limitation of our study is the absence of direct immunological profiling, which would provide valuable insights into host–virus–microbiome interactions. However, we addressed this in part by integrating surrogate markers such as STI history, smoking, and contraceptive use. We have now acknowledged this limitation explicitly and emphasize the need for future studies to include immune status assessment.

We acknowledge the absence of complete metadata for the pre-cancerous lesion group as a limitation of our study. This was primarily due to inconsistent data availability at the time of sample collection. Given the clinical importance of this transitional stage, future studies should prioritize comprehensive data collection for this group to enable more robust comparative analyses.

Awareness of the vaginal microbiome’s role in women’s health is growing, and researchers are now combining laboratory and clinical studies to develop a clear, standardized way to assess vaginal ecosystem health. However, we still do not fully understand how shifts in the vaginal flora drive HPV infection and the development of cervical pre-cancerous lesions. In this first study of its kind in Morocco, we have mapped these relationships in detail and identified new microbial and functional markers that could help to track treatment responses, predict patient outcomes, and clarify how HPV infection leads to cervical disease.

## 5. Conclusions

The present study characterizes the cervico-vaginal microbiome of Moroccan women across healthy, pre-cancerous, and post-treatment stages, showing that the lesion stage, rather than HPV status, drives the loss of *Lactobacillus*, the expansion of anaerobic and Gram-negative taxa, and shifts in nitrogen and sulfur metabolism.

## Figures and Tables

**Table 1:** Participants’ demographics and clinical characteristics.

Characteristic	Cases (*n* = 104)	Controls (*n* = 100)	Pre-Cancer (*n* = 39)
Age > 56 years	64 (60.9%)		MD
Age < 41 years		48 (48.0%)	
Married	61 (58.5%)	78 (78.0%)	
Insured	89 (84.8%)	99 (99.0%)	
Urban/suburban residence	68 (64.6%)	90 (90.0%)	
Illiterate	77 (73.8%)		
Higher education		65 (65.0%)	
Menarche ≤ 12 years	25 (23.8%)	51 (51.0%)	
Post-menopausal	94 (90.2%)	35 (35.0%)	
Early sexual debut (<18 y)	77 (74.0%)	26 (25.8%)	
Partner monogamy	55 (52.6%)	93 (92.7%)	
Prior STIs	42 (40.2%)	25 (25.0%)	
Smoking	38 (36.5%)	15 (15.0%)	
FIGO stage I	17 (16.6%)		
FIGO stage II	55 (53.3%)		
FIGO stage III	25 (23.8%)		
FIGO unclassified	7 (7.1%)		
HPV16	62 (59.6%)		
HPV53	15 (14.8%)		
HPV18	7 (6.4%)		
HPV83	2 (2.1%)		
HPV31	2 (2.1%)		
HPV89	2 (2.1%)		
HPV66	2 (2.1%)		
HPV62	2 (2.1%)		
HPV16/18 positive			39 (100%)

## Data Availability

The data presented in this study are available on request from the corresponding author due to ethical restrictions related to patient confidentiality and the sensitive nature of clinical and genetic information. Access will be granted to qualified researchers upon reasonable request and approval by the local ethics committee.

## Supplementary Materials

The following supporting information can be downloaded at: https://www.mdpi.com/article/10.3390/microorganisms13081884/s1. Figure S1: Phylum-level shifts in the cervico-vaginal microbiome revealed by volcano analysis; Figure S2: Co-occurrence network diagram of core vaginal microbiota across disease and HPV status; Table S1: Multiple logistic regression analysis of factors associated with clinical group status (reference group: Healthy Controls).
