# Supplementary material for: Cervico-Vaginal Microbiome Dynamics Across HPV-Driven Lesion Stages in Moroccan Women
Source: Microorganisms. 2025 Aug 13;13(8):1884. doi: 10.3390/microorganisms13081884 (PMC12388290; doi:10.3390/microorganisms13081884)
Supplement: Supplementary file 1 [file microorganisms-13-01884-s001.zip › microorganisms-3786098-supplementary.pdf]

# Cervico-Vaginal Microbiome Dynamics Across HPV-Driven Lesion Stages in Moroccan Women

Malika ALLALI *[1,2], Khaoula ERRAFII *[3], Rachid EL FERMI [3] ; Karima FICHTALI[4], Sanaa EL MAJJAOUI[4], Adil El GHANMI [4], Hicham EL FAZAZI [5], Najib AL IDRISSI[6] , Bouchra GHAZI [4], Youssef BAKRI [2] , Hassan GHAZAL [6,7,], Salsabil HAMDI [1$].

1: Virology and Public Health Laboratory, Institut Pasteur du Maroc, Casablanca Morocco.

2: Laboratory of Human Pathologies Biology, Department of Biology, Faculty of Sciences, University Mohammed V, Rabat, Morocco.

3: African Genome Center, Mohamed IV Polytechnic University, Benguerir 43151, Morocco.

4: Department of Gynecology and Obstetrics, Mohammed VI International University Hospital, Bouskoura, Morocco.

5: Fertility Center Cheikh Zaid International University Hospital, Abulcasis International University of Health Sciences, Rabat, Morocco.

6: Laboratory of Genomics, Genetics, Epigenetics, Precision and Predictive Medicines (PerMed), Faculty of Medicine, Mohammed VI University of Sciences and Health, Casablanca, Morocco.

7: Royal Institute of Managerial Training, Department of Sports Sciences, Sale, Morocco

**$: corresponding author:** salsabil.hamdi@pasteur.ma

***: equal contribution**

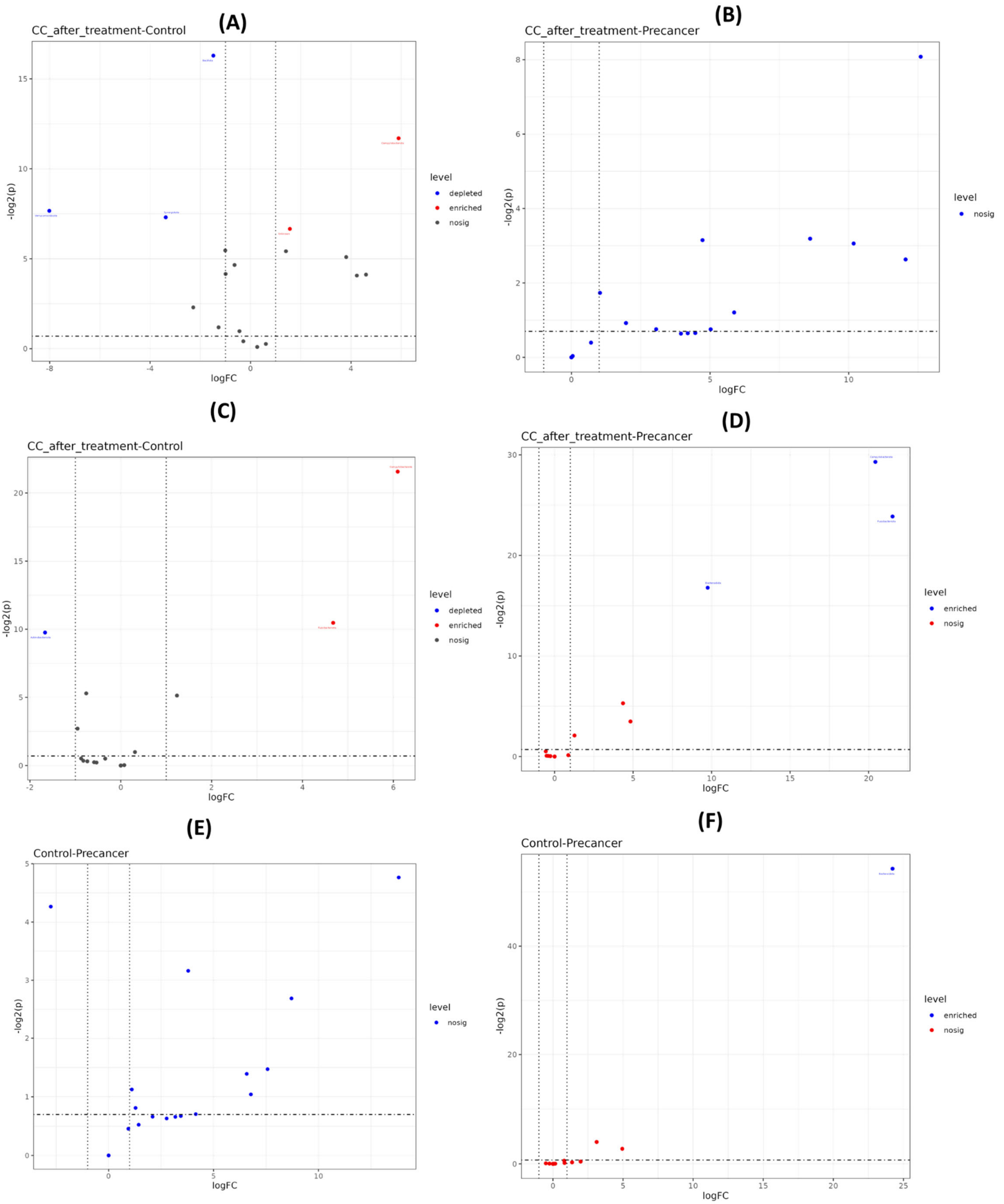


**S1.** Phylum-level shifts in the cervico-vaginal microbiome revealed by volcano analysis.

(A): Comparison of post-treatment cervical cancer cases versus healthy controls. (B): Comparison of post-treatment cervical cancer cases versus high-grade precancerous lesion samples. (C): Comparison of healthy controls versus high-grade precancerous lesion samples. (D): Comparison of high-grade precancerous lesion samples versus healthy controls. (E): Comparison of high-grade precancerous lesion samples versus post-treatment cervical cancer cases. (F): Comparison of healthy controls versus post-treatment cervical cancer cases.

**(A)**

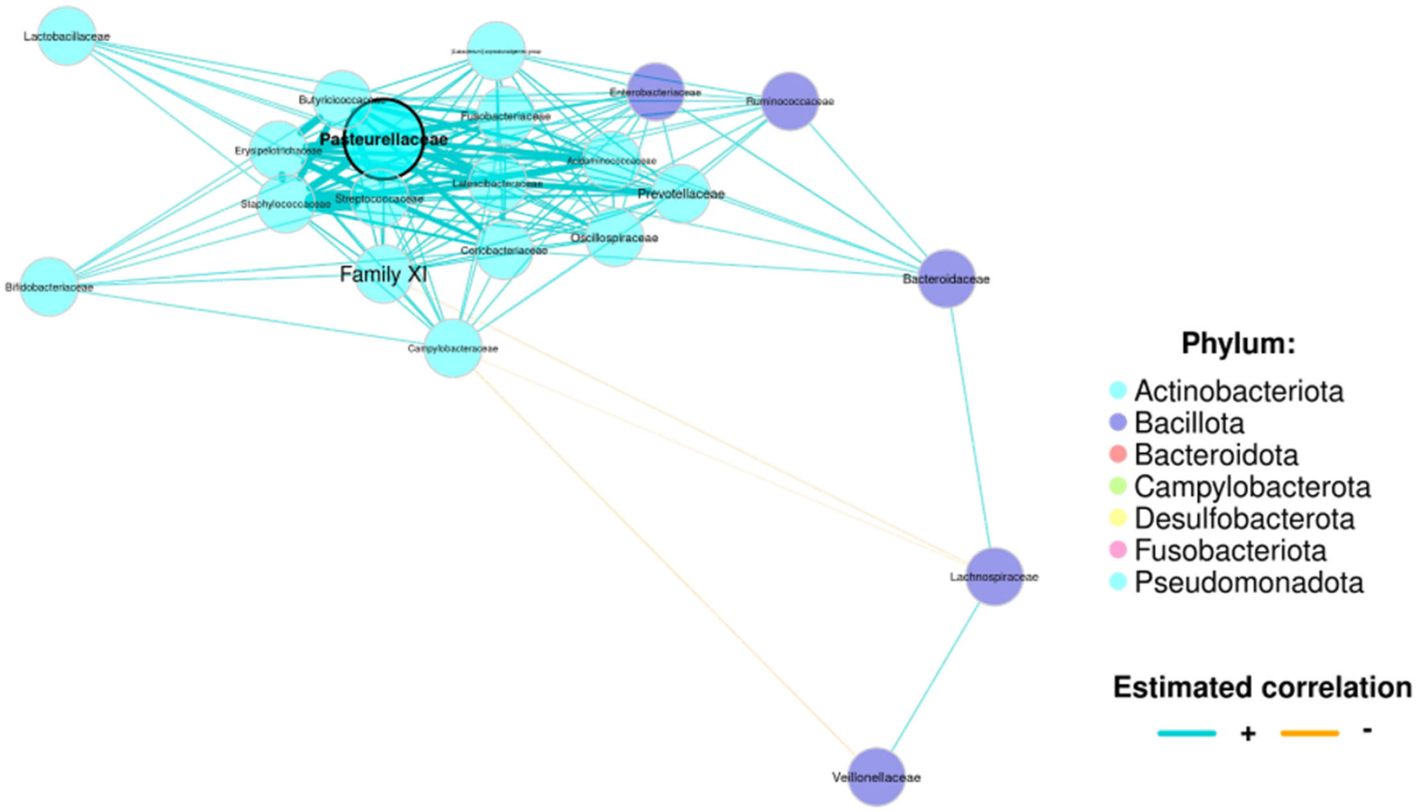

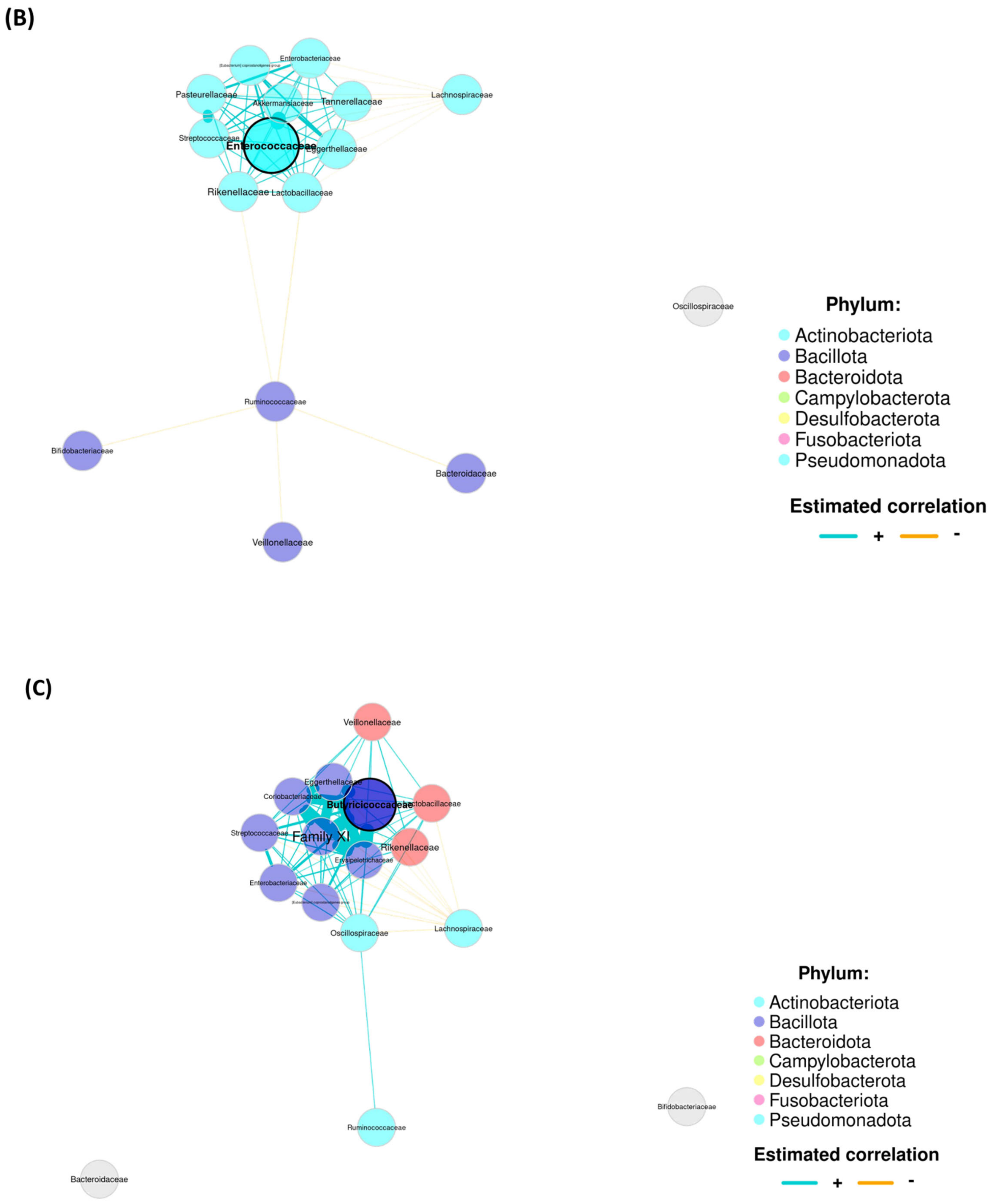
(B)
Enterobacteriaceae
Pasteurellaceae
Akkermansiaceae
Tannerellaceae
Lachnospiraceae
Streptococcaceae
Enterococcaceae
Eggerthellaceae
Rikenellaceae
Lactobacillaceae
Oscillospiraceae
Ruminococcaceae
Bifidobacteriaceae
Bacteroidaceae
Veillonellaceae
Phylum:
Actinobacteriota
Bacillota
Bacteroidota
Campylobacterota
Desulfobacterota
Fusobacteriota
Pseudomonadota
Estimated correlation
+
-
(C)
Veillonellaceae
Eggerthellaceae
Coriobacteriaceae
Butyricicoccaceae
Lactobacillaceae
Streptococcaceae
Family XI
Rikenellaceae
Erysipelotrichaceae
Enterobacteriaceae
Oscillospiraceae
Lachnospiraceae
Ruminococcaceae
Bifidobacteriaceae
Bacteroidaceae
Phylum:
Actinobacteriota
Bacillota
Bacteroidota
Campylobacterota
Desulfobacterota
Fusobacteriota
Pseudomonadota
Estimated correlation
+
-

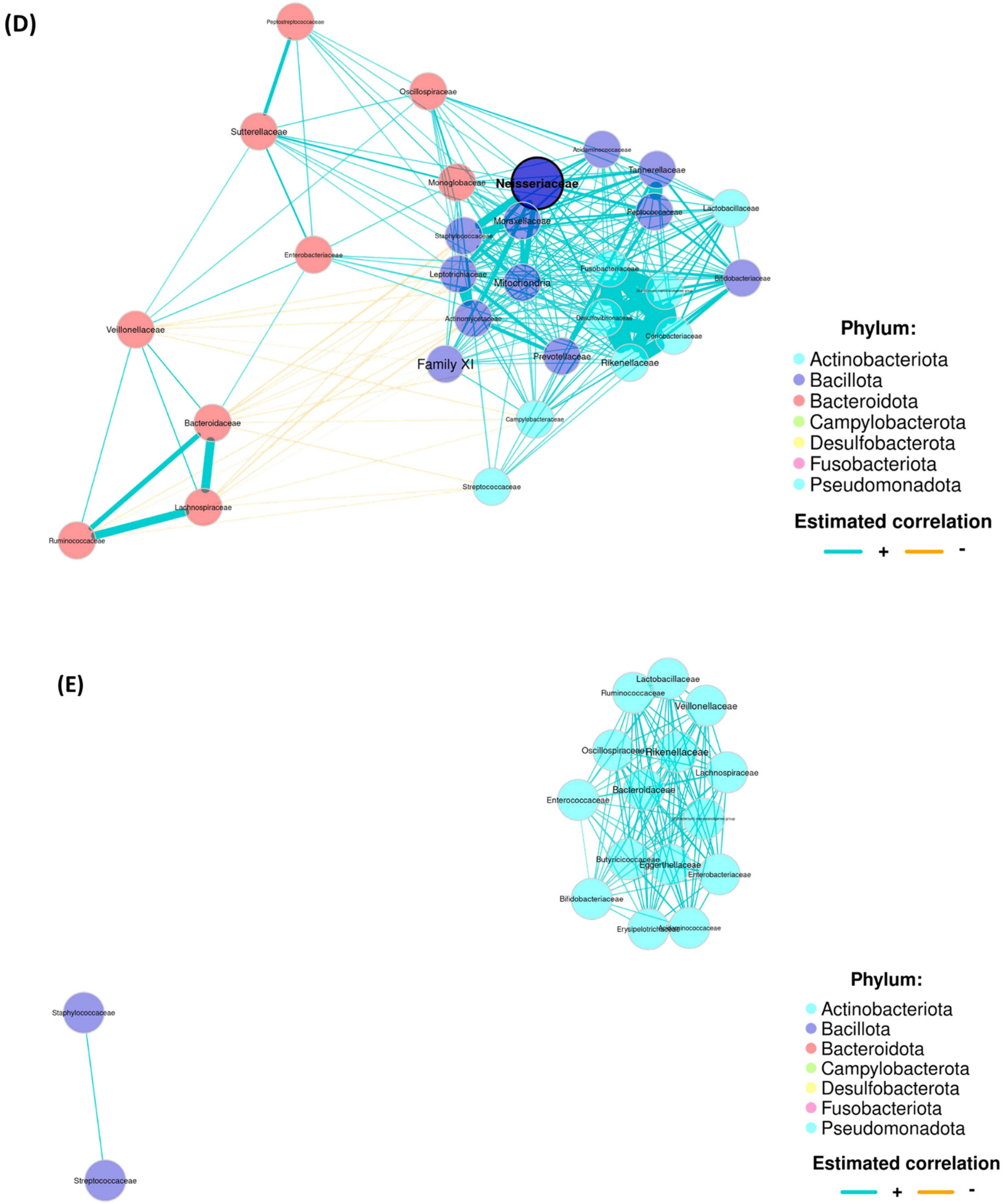
(D)
Peptostreptococcaceae
Oscillospiraceae
Sutterellaceae
Monoglobaceae
Neisseriaceae
Acidaminococcaceae
Tannerellaceae
Lactobacillaceae
Peptococcaceae
Moraxellaceae
Staphylococcaceae
Enterobacteriaceae
Fusobacteriaceae
Leptotrichiaceae
Mitochondria
Bifidobacteriaceae
Veillonellaceae
Actinomycetaceae
Desulfovibrionaceae
Coriobacteriaceae
Family XI
Prevotellaceae
Rikenellaceae
Campylobacteraceae
Bacteroidaceae
Streptococcaceae
Lachnospiraceae
Ruminococcaceae
Phylum:
Actinobacteriota
Bacillota
Bacteroidota
Campylobacterota
Desulfobacterota
Fusobacteriota
Pseudomonadota
Estimated correlation
+
-
(E)
Lactobacillaceae
Ruminococcaceae
Veillonellaceae
Oscillospiraceae
Rikenellaceae
Lachnospiraceae
Bacteroidaceae
Enterococcaceae
Butyricicoccaceae
Eggerthellaceae
Enterobacteriaceae
Bifidobacteriaceae
Staphylococcaceae
Streptococcaceae
Phylum:
Actinobacteriota
Bacillota
Bacteroidota
Campylobacterota
Desulfobacterota
Fusobacteriota
Pseudomonadota
Estimated correlation
+
-

**S2.** Co-occurrence network diagram of core vaginal microbiota across disease and HPV status. (A): Network in cervical cancer cases after radiochemotherapy. (B): Network in healthy control samples. (C): Network in high-grade precancerous lesion samples. (D): Network in HPV-positive women. (E): Network in HPV-negative women.

S3: Multiple logistic regression analysis of factors associated with clinical group status (reference group: Healthy Controls)

| **Predictor Variable** | **Odds Ratio (OR)** | **95% Confidence Interval** | **p-value** |
|---|---|---|---|
| Age > 56 years | 2.85 | 1.54–5.28 | 0.001 ** |
| Married | 0.52 | 0.30–0.91 | 0.023 * |
| Illiterate | 2.97 | 1.54–5.72 | 0.001 ** |
| Post-menopausal | 3.14 | 1.62–6.10 | 0.001 ** |
| Early sexual debut (<18 y) | 4.67 | 2.43–9.00 | <0.001 ** |
| Prior STIs | 1.95 | 1.02–3.72 | 0.043 * |
| Smoking | 3.32 | 1.63–6.78 | 0.001 ** |
| Urban/Suburban residence | 0.41 | 0.19–0.90 | 0.027 * |
